# Predicting activities of daily living (ADL) Decline in Alzheimer’s Disease: The Combined Impact of Motor Performance and White matter hyperintensities (WMH)

**DOI:** 10.1192/j.eurpsy.2026.10664

**Published:** 2026-07-31

**Authors:** J. H. Park, H. J. Yang

**Affiliations:** Psychiatry, Jeju National University Hospital, Jeju, Korea, Republic Of

## Abstract

**Introduction:**

Alzheimer’s disease (AD) leads to progressive decline in cognitive and functional abilities, with activities of daily living (ADL) serving as a key indicator of disease severity. Gait speed and grip strength are recognized as markers of physical and cognitive health, but their combined and mediating roles in AD remain insufficiently studied.

**Objectives:**

This study aims to investigate the effects of gait speed and grip strength on ADL and their mediating roles between brain pathology and ADL in patients with AD.

**Methods:**

We retrospectively analyzed 150 patients aged ≥65 years diagnosed with AD at Jeju National University Hospital (2017–2024). Data included gait speed, grip strength, ADL, Clinical Dementia Rating Sum of Box (CDR-SB), CERAD neuropsychological assessments, and brain MRI measures (hippocampal volume and white matter hyperintensities (WMH)). Multiple regression and path analyses were conducted to examine the direct, interactive, and mediating effects of gait speed and grip strength on ADL and dementia severity.

**Results:**

Multivariate regression analysis revealed that slower gait speed (β = −0.28, p = 0.024) and weaker grip strength (β = −0.23, p = 0.012) were independently associated with lower ADL performance. A significant interaction effect between gait speed and grip strength was observed, explaining additional variance in ADL decline beyond individual effects (ΔR^2^ = 0.06, p < 0.01). Increased WMH volume was significantly correlated with slower gait speed (β = −0.30, p < 0.001) and weaker grip strength (β = −0.11, p = 0.042). Path analysis further demonstrated that motor performance partially mediated the relationship between WMH volume and ADL decline (β= 0.18, 95% CI: 0.11–0.26).

**Conclusions:**

This study demonstrates that gait speed and grip strength serve as important independent predictors of functional decline in Alzheimer’s disease, with their combined assessment providing enhanced predictive value for ADL deterioration. Motor performance serves as a critical link between brain pathology and functional outcomes, highlighting the importance of incorporating motor assessments into AD management and progression monitoring.This study aims to investigate the effects of gait speed and grip strength on ADL and their mediating roles between brain pathology and ADL in patients with AD.

**Disclosure of Interest:**

None Declared

